# Whole-genome sequencing analysis in families with recurrent pregnancy loss: A pilot study

**DOI:** 10.1371/journal.pone.0281934

**Published:** 2023-02-17

**Authors:** Tsegaselassie Workalemahu, Cecile Avery, Sarah Lopez, Nathan R. Blue, Amelia Wallace, Aaron R. Quinlan, Hilary Coon, Derek Warner, Michael W. Varner, D. Ware Branch, Lynn B. Jorde, Robert M. Silver

**Affiliations:** 1 Department of Obstetrics and Gynecology, University of Utah Health, Salt Lake City, Utah, United States of America; 2 Department of Human Genetics, University of Utah, Salt Lake City, Utah, United States of America; 3 Intermountain Healthcare, Maternal-Fetal Medicine, Salt Lake City, Utah, United States of America; 4 Department of Biomedical Informatics, University of Utah, Salt Lake City, Utah, United States of America; 5 Department of Psychiatry, University of Utah, Salt Lake City, Utah, United States of America; 6 DNA Sequencing Core, University of Utah, Salt Lake City, Utah, United States of America; Manisa City Hospital, TURKEY

## Abstract

One to two percent of couples suffer recurrent pregnancy loss and over 50% of the cases are unexplained. Whole genome sequencing (WGS) analysis has the potential to identify previously unrecognized causes of pregnancy loss, but few studies have been performed, and none have included DNA from families including parents, losses, and live births. We conducted a pilot WGS study in three families with unexplained recurrent pregnancy loss, including parents, healthy live births, and losses, which included an embryonic loss (<10 weeks’ gestation), fetal deaths (10–20 weeks’ gestation) and stillbirths (≥ 20 weeks’ gestation). We used the Illumina platform for WGS and state-of-the-art protocols to identify single nucleotide variants (SNVs) following various modes of inheritance. We identified 87 SNVs involving 75 genes in embryonic loss (n = 1), 370 SNVs involving 228 genes in fetal death (n = 3), and 122 SNVs involving 122 genes in stillbirth (n = 2). Of these, 22 *de novo*, 6 inherited autosomal dominant and an X-linked recessive SNVs were pathogenic (probability of being loss-of-function intolerant >0.9), impacting known genes (e.g., *DICER1*, *FBN2*, *FLT4*, *HERC1*, and *TAOK1*) involved in embryonic/fetal development and congenital abnormalities. Further, we identified inherited missense compound heterozygous SNVs impacting genes (e.g., *VWA5B2*) in two fetal death samples. The variants were not identified as compound heterozygous SNVs in live births and population controls, providing evidence for haplosufficient genes relevant to pregnancy loss. In this pilot study, we provide evidence for *de novo* and inherited SNVs relevant to pregnancy loss. Our findings provide justification for conducting WGS using larger numbers of families and warrant validation by targeted sequencing to ascertain causal variants. Elucidating genes causing pregnancy loss may facilitate the development of risk stratification strategies and novel therapeutics.

## Introduction

Pregnancy loss is a common obstetric complication leading to significant economic and emotional burden for affected families and the health care system [1]. Women experiencing pregnancy loss are at increased risk of its recurrence, as well as other obstetric complications in subsequent pregnancies [2–4]. Recurrent pregnancy loss occurs in 1–2% of couples who are trying to conceive [5, 6]. Recurrent pregnancy loss is commonly defined by the American Society of Reproductive Medicine as ≥ 2 pregnancy losses [7], and because the etiologies of pregnancy loss vary across gestational age, more specific characterizations of losses by gestational age have been recommended [8]. Thus, pregnancy loss can be divided into three epochs: embryonic loss (<10 weeks’ gestation), fetal death (10–20 weeks’ gestation) and/or stillbirth (≥20 weeks’ gestation).

Though known and suspected causes of recurrent pregnancy loss include autoimmune, endocrine, uterine, and genetic abnormalities, over half are not currently explained by these mechanisms [9–11]. Among genetic abnormalities, the most clearly associated with recurrent pregnancy loss is parental balanced translocation [12]. However, this abnormality is found in fewer than 5% of couples with recurrent pregnancy loss [13, 14]. Embryonic losses (<10 weeks) are often due to spontaneously-occurring aneuploidy which result from errors in maternal meiosis [7]. Such cases are identified by karyotype but often have a low recurrence risk [15].

Many previous studies of pregnancy loss did not distinguish gestational ages of the losses and focused on sporadic losses <10 weeks [7, 16]. However, systematic evaluation of unexplained embryonic loss, fetal death and stillbirth cases is critical to identify genetic abnormalities that are not detected by karyotype and may influence specific developmental epochs. Whole-genome sequencing (WGS) allows identification of previously unrecognized genetic abnormalities (e.g., copy number changes, single gene mutations, single nucleotide variants [SNVs] and/or structural variants [SVs]) that may cause unexplained pregnancy loss [10]. Few studies included DNA from parents, losses, and live births. The power of WGS technology can be further amplified by examining DNA from family pedigrees to clarify autosomal-dominant transmission of risk alleles and prove whether variants appeared in the germline of the probands as *de novo*, which will be critical for interpretation and determination of genetic causes of recurrent and sporadic pregnancy loss.

Therefore, we conducted a pilot WGS study of four families with several unexplained pregnancy losses, which included embryonic loss, fetal death and stillbirth. We applied best practice standards of WGS and analyses to identify variants using DNA from couples and their products of conception (pregnancy losses and live births). We hypothesized that pathogenic SNVs and/or SVs that may be inherited or occur *de novo* in the offspring will be relevant to the losses.

## Materials and methods

### Description of study participants

Our pilot study included patients who received care at the University of Utah and had suffered at least two pregnancy losses with at least one uncomplicated live birth and in whom evaluation for accepted causes of sporadic and recurrent pregnancy loss had proven negative [7, 17]. Not all cases had complete evaluations which were performed at the discretion of the providers. This study was approved by the Institutional Review Board (IRB) of the University of Utah (IRB #: 00055018; date: 3/13/2019). All participants completed a written and verbal informed consent process, conducted with research staff, prior to their initial enrollment. After signature capture, consenting participants were provided with a copy of the signed IRB-approved consent form for their personal records. Children who were under age 18 were consented with an assent and parental permission document. All consents included a statement that withdrawal from the study at any time was allowed. Participants were made aware that they will not be provided with the results from the sequencing except in the case of incidental findings that are medically actionable. Participants were notified that they would have the opportunity to decline the return of these incidental findings on the consent form and again prior to their release. All data were fully anonymized. Pregnancy losses in these patients included embryonic losses (<10 weeks), fetal deaths (10–20 weeks) and/or stillbirths (≥20 weeks). Data regarding medical and reproductive examinations (e.g., uterine abnormalities, parental karyotypic and chromosomal microarray abnormalities, endocrinopathies including diabetes) were obtained by medical record abstraction and patient interview. In this pilot study, we included four families with available biospecimens from parents their products of conception (pregnancy losses and live births) for DNA sequencing.

### Data and sample collection

Couples received saliva sample and buccal swab kits to collect cells for DNA sampling with instructions along with a brief questionnaire for demographic data collection. Research team and obstetricians examined patient clinical and demographic data and entered the data in REDcap. Couples provided spit saliva and buccal saliva from their live-born children. Placenta samples from pregnancies that resulted in fetal demise were processed by pathology within three days of delivery. One family with a known aneuploid stillbirth (Family 3) was included since they had five unexplained losses (**Table 1**). Placentas were processed using clinical protocols for placental pathology, and samples were obtained from formalin fixed and paraffin embedded (FFPE) blocks and stored at room temperature. In some cases, samples were collected for research only. In these cases, placentas were washed and dissected from fetal villi and maternal decidual tissue to ensure sampling of fetal tissue. Tissue from these samples were divided into aliquots and stored at -80°C.

**Table 1:** Characteristics of study participants.

	Family 1	Family 2	Family 3	Family 4
**Maternal**				
Age at first pregnancy, years	26	34	25	26
Genotype inferred Race/Ethnicity	Non-Hispanic White	Hispanic	Non-Hispanic White	Non-Hispanic White
Body-mass-index kg/m2	26.6	27.1	19.1	32.3
**Paternal**				
Age at first pregnancy, years	34	36	35	35
Genotype inferred Race/Ethnicity	Non-Hispanic White	Non-Hispanic White	Non-Hispanic White	Non-Hispanic White
**Pregnancy outcome and samples**				
1st Pregnancy	Male live-birth (>37 weeks) buccal swab ^a^	Male live-birth (>37 weeks) buccal swab ^a^	Female live-birth (>37 weeks) buccal swab ^a^	Male live-birth (>37 weeks) buccal swab ^a^
2nd Pregnancy	Male stillbirth (20 weeks) frozen placenta ^a^	Unknown sex fetal death (13 weeks 6 days)	Male stillbirth (20–40 weeks) FFPE placenta ^a^	Female live-birth (>37 weeks) buccal swab ^a^
3rd Pregnancy	Female embryonic loss (6 weeks 6 days)	Male live-birth (>37 weeks) buccal swab ^a^	Unknown sex pre-embryonic loss (5 weeks 6 days) FFPE placenta ^a^	Female live-birth (>37 weeks) buccal swab ^a^
4th Pregnancy	Male fetal death (15 weeks 6 days) FFPE placenta ^a^	Unknown sex stillbirth (20–23 weeks) FFPE placenta ^a^	Unknown sex pre-embryonic loss (6–9 weeks)	Unknown sex embryonic loss (6–9 weeks)
5th Pregnancy	Male live-birth (>37 weeks) buccal swab ^a^	Unknown sex embryonic loss (8 weeks 6 days)	Female live-birth (>37 weeks)	Male fetal death (17 weeks 6 days) FFPE placenta ^a^
6th Pregnancy	Male fetal death (13–20 weeks) FFPE placenta ^a^	Unknown sex embryonic loss (9 weeks 6 days)	Female live-birth (>37 weeks) buccal swab ^a^	Unknown sex stillbirth (20–23 weeks)
7th Pregnancy	Male fetal death (13 weeks 6 days)	Unknown sex embryonic loss (7 weeks 6 days)	Unknown sex fetal death (14 weeks 6 days)	Male fetal death (18 weeks 6 days) FFPE placenta ^a^
8th Pregnancy	Male fetal death (13 weeks 6 days) FFPE placenta ^a^	-	Unknown sex live-birth (>37 weeks)	-
Birth defects	No	No	Yes	No
Thyroid Disease	Yes	No	No	Yes
Karyotype testing	Normal all pregnancies	-	Abnormal 2nd pregnancy	-
Microarray	Normal 7th pregnancy	-	Not ordered	-
**Products of conception with WGS data**				
Embryonic loss, N	-	-	1	-
Fetal death, N	3	-	-	2
Stillbirth, N	1	1	1	1
Live-birth, N	2	2	3	3

^a^Obtained DNA from samples for WGS

### DNA extraction and whole-genome sequencing

DNA from saliva and FFPE samples was purified and extracted using Qiagen Kit (Qiagen Systems) and Promega Kit, respectively. WGS libraries were prepared for Illumina 150bp paired-end reads sequencing using the NEBNext Ultra II DNA Library Prep Kit protocols. All libraries were sequenced on the Novaseq 6000 platform (Illumina, San Diego, CA, USA) using standard protocols. Whole-genome analysis was performed by the Utah Center for Genomic Discovery (UCGD) at the University of Utah. Germline SNVs and SVs for each sample (22 samples total) were detected following a Genome Analysis Tool Kit (GATK) best practices equivalent workflow for variant detection [18].

### Variant detection and quality control of WGS

Variant detection and quality control protocol details are provided in **S1 File**. Variant detection methods were tuned to detect low-frequency mutations (gnomAD allele frequency [AF]<0.001) to explore and compare germline variants in protein coding regions (potentially impactful variants) across samples.

### Variant prioritization and selection of candidate genes relevant to pregnancy loss

We used Slivar [19] to prioritize and filter variants based on modes of inheritance (e.g., compound heterozygous, *de novo*, autosomal dominant and x-linked recessive). Slivar integrates population allele frequencies from the Trans-Omics for Precision Medicine (TopMED) [20] and spliceAI scores into a comprehensive variant filtering strategy to identify candidate genes [19]. While autosomal dominant and compound heterozygous variants may include *de novo*, we prioritized on inherited variants separately that may be relevant to non-sporadic losses. Details on variant prioritization and exploratory analyses of variants relevant to recurrent pregnancy loss are provided in **S1 File** and **S1 Table**. We evaluated SNVs across the families by modes of inheritance and highest impact on genes (in-frame deletion/insertion, missense [nonsynonymous], frameshift, stop gained, splice region).

Given the potential for identifying false positive germline SNVs due to DNA quality (e.g., prioritization of false positive autosomal dominant SNVs that differ by orders of magnitude from SNVs following other modes of inheritance [19]) and overwhelming majority of variants of unknown significance, we applied several approaches to interpret our main findings. First, we selected SNVs identified in any of the pregnancy losses but not live births within our data to interpret candidate genes relevant to recurrent pregnancy loss. Second, we interpreted inherited rare (AF < .001) compound heterozygous SNVs, autosomal recessive variants, where both parents are heterozygous for the variant and the affected offspring receives two copies. We prioritized inherited compound heterozygous SNVs that were identified in losses within our data but found as compound heterozygous SNVs in healthy controls (gnomAD [21]) to highlight variants in haplosufficient genes relevant to embryonic/fetal lethality. Third, among SNVs that were identified in any of the pregnancy losses, we selected pathogenic SNVs (SNVs with pLI>0.90 and LOEUF<0.36) to highlight potentially damaging variants in candidate genes. Finally, we selected SNVs in genes that were involved in pregnancy loss-relevant phenotypes/diseases (e.g., embryonic/fetal death and developmental abnormalities [22–24]) to interpret candidate genes. Analyses were performed using Slivar and R, utilizing resources and support from the Center for High Performance Computing at the University of Utah.

## Results

### Summary of study participants

Study participants included four families with 3–6 losses and 2–4 live births (**Table 1**). Participants’ maternal and paternal ages ranged between 25–34 and 34–36 years, respectively. All maternal and paternal participants self-identified as non-Hispanic White. Genetic ancestry inferred from the genotype of the participants suggested White/Hispanic, i.e., admixed Americans for the Family 2 mother and White/non-Hispanic, i.e., Western European ancestry for all other participants. Family 3 had an abnormal karyotype stillborn fetus in their second pregnancy. Samples were available from an embryonic loss at 5 weeks and 6 days (Family 3), fetal deaths at 15 weeks and 6 days, 13–20 weeks, and 13 weeks and 6 days (Family 1), 17 weeks and 6 days, and 18 weeks and 6 days (Family 4), and stillbirths at 20 weeks (Family 1), 20–23 weeks (Family 2) and 20–40 weeks (Family 3). Samples from live births (n = 10 from four families) were healthy babies born after 37 weeks.

### SNVs relevant to recurrent pregnancy loss

**Fig 1:**
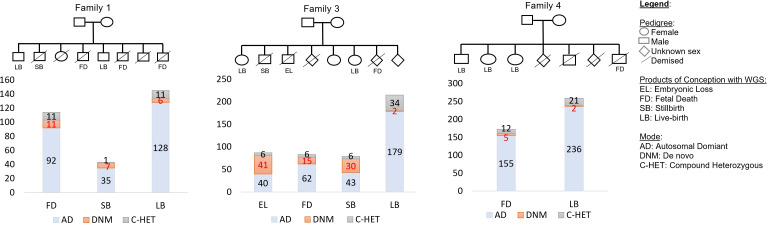
Number of SNVs by modes of inheritance and products of conception.

After removing poor DNA quality samples and samples failing sex-check (five pregnancy losses samples and one family), 3,211,893 SNVs remained for further analysis. Finally, 28,485 impactful SNVs (i.e., missense, frameshift, insertion/deletion, stop gained/retained, and splice region) in all samples from the products of conception (n = 16 in three families) were prioritized by Slivar. Using samples that passed quality control (n = 16 in three families; **S1 File**), we identified 87 SNVs involving 75 genes in an embryonic loss sample, 370 SNVs involving 228 genes in three fetal death samples, and 122 SNVs involving 122 genes in two stillbirth samples (**Fig 1** and **Table 2**). In Family 1, the SNVs included 11 inherited compound heterozygous, 11 *de novo* and 92 inherited autosomal dominant in the fetal death cases, and 1 inherited compound heterozygous, 7 *de novo* and 35 inherited autosomal dominant in stillbirth cases (**Fig 1**). In addition, the SNVs in Family 2 included 6 inherited compound heterozygous, 41 *de novo* and 40 inherited autosomal dominant in the embryonic loss case, 6 inherited compound heterozygous, 15 *de novo* and 62 inherited autosomal dominant in the fetal death case, and 6 inherited compound heterozygous, 30 *de novo* and 43 inherited autosomal dominant in the stillbirth case. Further, the SNVs in Family 4 included 12 inherited compound heterozygous, 5 *de novo* and 155 inherited autosomal dominant in the fetal death case. Several SNVs identified in our data impact genes that were known to be involved in the development of the embryo and fetus, and congenital abnormalities (e.g., *DICER1* [25], *FBN2* [22], *FLT4* [26], *HERC1* [27, 28], and *TAOK1* [29]).

**Table 2:** Number of SNVs (and genes) in losses.

Family	Embryonic Loss		Fetal Death		Stillbirth	
	SNVs (N)	Genes (N)	SNVs (N)	Genes (N)	SNVs (N)	Genes (N)
Family 1	-	-	114	89	43	44
Family 3	87	75	83	75	79	68
Family 4	-	-	173	127	-	-
**Total** ^a^	87	75	370	228	122	112

^a^Total counts account for overlapping SNVs/genes across the samples.

**Note**: The crude counts also represent SNVs that may be present in live births.

Among the SNVs we identified, 29 SNVs are predicted as pathogenic (pLI>0.9; LOUEF<0.36), impacting 27 genes, several of which are involved in known diseases (**S2 Table**). Specifically, we identified three autosomal dominant and three *de novo* pathogenic SNVs in fetal death and stillbirth from Family 1, one inherited autosomal dominant and sixteen *de novo* pathogenic SNVs in embryonic loss, fetal death and stillbirth from Family 3, and one inherited autosomal dominant, one X-linked recessive and three *de novo* pathogenic SNVs in fetal death from Family 4. Given the counts of *de novo* SNVs that are higher in losses than live births, we provided details, which included a table of loss-of-function *de novo* SNVs by pathogenicity and gene impact and exploratory *de novo* enrichment analysis (**S1 File** and **S3 Table**). *De novo* SNVs were predominantly missense (nonsynonymous) followed by frameshift, splice region, *in-frame* deletion/insertion and stop gained. The observed mean *de novo* loss-of-function SNVs in pregnancy losses was higher than that of the expected (2.0 vs 0.2; p-value = 0.01). Moreover, the SNVs were enriched in >1 protein altering genes (p-value<0.001).

Furthermore, among inherited compound heterozygous SNVs we identified, four SNVs in three genes (*TM2D1*, *MUC16*, *VWA5B2*) were identified in fetal death from Family 1 but not in any of the live births (**Table 3**). The SNVs were not observed as homozygotes in healthy controls, highlighting their potential relevance to pregnancy loss in our samples. Finally, we conducted exploratory analyses to confirm and validate our findings, which included exploratory SNV rates comparison (**S1 Table**), rare-variant association, and Sanger sequencing analyses. The methods and summary of results based on our exploratory analyses are provided in **S1 File.**

**Table 3:** Compound heterozygous SNVs identified in losses but not live births and gnomAD controls.

Family	SNV	cDNA Position	Highest impact	Gene	Nomenclature	Biological function ^a^	Associated Diseases
Family 1 Male fetal death in the 6th pregnancy at 13–20 weeks’ gestation	chr1:61723786:T:C	178	Splice region	*TM2D1*	Beta-Amyloid Peptide Binding Protein	G protein-coupled receptor signaling pathway	Familial Alzheimer disease
	chr1:61725096:G:A	18	Missense				
	chr19:8939543:C:T	31616	Missense	*MUC16*	Mucin 16	Involved in cell adhesion	Ovarian cancer
	chr19:8953864:G:T	23110	Missense				
Family 1 Male fetal death in the 8th pregnancy at 13 weeks and 6 days gestation	chr3:184236380:T:C	759	Missense	*VWA5B2*	Von Willebrand factor A domain containing 5B2	-	Usher syndrome

^a^Gene Ontology (GO) terms

## Discussion

Our pilot WGS study identified 87 SNVs involving 75 genes in embryonic loss (n = 1), 370 SNVs involving 228 genes in fetal death (n = 3), and 122 SNVs involving 122 genes in stillbirth (n = 2) samples, as potentially related to pregnancy loss, across three families. The SNVs included twenty-two *de novo*, six inherited autosomal dominant and one X-linked recessive mutation(s) that had high pathogenicity scores (pLI>0.9; LOUEF<0.36). In addition, our findings for higher counts of *de novo* SNVs in losses compared with live births, excess of genes with >1 loss-of-function *de novo* SNVs (p-value = 0.01), and occurrence of multiple *de novo* events in a single gene in samples from losses, implicate *de novo* SNVs in the pathogenesis of pregnancy loss. Furthermore, several of the identified SNVs impact genes (e.g., *DICER1* [25], *FBN2* [22], *FLT4* [26], *HERC1* [27, 28], and *TAOK1* [29]) that were known to be involved in the development of the embryo and fetus, and that are associated with congenital abnormalities, highlighting the potential role of SNVs in phenotypes that may share a common pathway with recurrent pregnancy loss. Lastly, we identified inherited missense compound heterozygous SNVs impacting genes (e.g., *VWA5B2*) in two fetal death samples that were absent from live births and population controls, providing evidence for haplosufficient genes relevant to pregnancy loss.

Previous genetic studies of pregnancy loss are limited for several reasons, including (1) lack of access to paternal DNA samples, which would make interpretations difficult without distinguishing inherited form from *de novo* variants [30], (2) unavailability of pedigrees with products of conception from chromosomally normal losses and live-births, or (3) unavailability of high-quality data and protocols for DNA restoration and variant detection [31–33]. Loss-of-function risk variants and inherited variants in intolerant genes (i.e., genes that are critical for human development, conditions incompatible with life resulting in fetal demise) [16, 23, 34] were not identified, possibly due to limited sample size and focus on families with recurrent, rather than sporadic losses.

Recently, whole-exome sequencing of stillbirth in maternal-offspring duos was conducted to identify variants in intolerant genes that were impossible to ascertain with karyotype or microarray [23]. Though the study was limited in ascertaining *de novo* from inherited variants, due to unavailability of paternal DNA, genes were reported by the authors that are either lethal, known to cause disease, or increase stillbirth risk (e.g., *CCR5*, *FAT1*, *FLNB*, *INPP5K*, *MYO1C*, *PLOD2*). Importantly, these genes overlap with findings in our data. For example, we identified a *de novo* missense chr3:58141895:C:T in *FLNB* and an inherited autosomal dominant missense chr17:1471262:C:T in *MYO1C*, two previously described genes in the literature, in stillbirth in Family 3 and Family 1, respectively. *FLNB* (Filamin B) is known for its role in atelosteogenesis type 1, a genetic disease characterized by a severe short-limbed dwarfism that is lethal in the perinatal period [35]. *FLNB* binds to actin to form the branching network of filaments that makes up the cytoskeleton, and is involved in the development of the skeleton before birth [36]. Missense actin mutations in *FLNB* leading to atelosteogenesis type I [37] and lethal skeletal dysplasia or inhibition of ERK/MMP-2 and MMP-9 pathways that are critical for trophoblast invasion [38], may be possible mechanisms of potentially lethal *de novo FLNB* SNVs in stillbirth etiology [39]. Similarly, *MYO1C* (Myosin isoform C) encodes actin-based motor molecules involved in insulin and VEGFA-VEGFR2 signaling pathways and chromatin remodeling. Although inherited mutations in *MYO1C* have been described for deafness in humans and mice [40], *MYO1C*’s role in cytoskeletal development, similar to that of *FLNB*, suggests a potential mechanism for inherited lethal SNVs in stillbirth [36]. Given that the SNVs in *FLNB* and *MYO1C* genes were not identified in live births in our data, the findings warrant validation to confirm potentially lethal variants causing chromosomally normal stillbirths.

Recently, Kline *et*. *al*. similarly hypothesized that chromosomally normal losses are caused by rare variants in several different genes, some of which are incompatible with development to the fetal stage [22]. The authors reported damaging variants in several genes that are relevant to recurrent pregnancy loss, including *FBN2*. *FBN2* (Fibrillin 2) encodes peptide hormone placensin that is secreted by trophoblasts to promote trophoblast invasiveness [41]. Missense variants in *FBN2* are known to cause congenital contractual arachnodactyly [42]. Mechanisms of trophoblast invasion and congenital contractual arachnodactyly are described in embryonic development etiology [22]. Furthermore, a novel frameshift variant was previously found in a stillborn fetus [23]. In our study, we identified a *de novo* in-frame deletion involving *FBN2* in fetal death in Family 4 that was not identified in any of the live births across the families. Although Kline *et*. *al*. identified inherited compound heterozygous variants of *FBN2* in embryonic loss, our SV analysis in stillbirth in Family 4 (see **S1 File**) confirmed a *de novo* SV (chr5:128335405) impacting *FBN2*, suggesting variants disrupting the *FBN2* gene may be incompatible with development to the fetal stage. Thus, *FBN2* may be a potential candidate worth investigating in larger studies [43, 44].

Given the small participant sample with WGS data in our pilot study, it is noteworthy that we identified variants in several genes (e.g., *DICER1* [25], *FBN2* [22], *FLT4* [26], *HERC1* [27, 28], and *TAOK1* [29]) that were previously identified by genetic studies of pregnancy loss. *DICER1* is essential for the synthesis and biogenesis of miRNAs [36]. Though we identified a *de novo* frameshift SNV in stillbirth, other polymorphisms in *DICER1* were previously shown to be associated with spontaneous miscarriage before 20 weeks’ gestation [45] and recurrent pregnancy loss before 14 weeks’ gestation [46]. Suggested mechanisms of *DICER1* include decidualization of endometrial stroma, which are critical trophoblast invasion and placental function [25, 46, 47]. Failures in trophoblast invasion and placental formation can compromise embryonic development and lead to stillbirth [48]. In addition, *FLT4* (Fms Related Receptor Tyrosine Kinase 4) encodes the vascular endothelial growth factor receptor 3 (VEGFR-3) receptor [49] and is described for its role in congenital heart disease. Vascular endothelial growth factor promotes angiogenesis at the early embryonic stage of pregnancy [50]. Vascular endothelial growth factor gene polymorphisms are associated with recurrent pregnancy loss < 20 weeks’ gestation [51], suggesting that the autosomal dominant missense SNV we identified in *FLT4* gene in a fetal death in Family 3 may be relevant to pregnancy loss. Furthermore, *HERC1* (HECT And RLD domain containing E3 ubiquitin protein ligase family member 1) is a functional gene for ubiquitin-protein transferase activity and maintenance of the cerebellar structure [52]. Mutations in *HERC1* in the mouse may be lethal *in utero* [52]. In our study, we identified a *de novo* SNV impacting *HERC1* in embryonic loss in Family 3 as stop gained, missense and frameshift mutation, suggesting the potential relevance of lethal *SNVs* in embryonic loss. Lastly, *TAOKI* (thousand and one amino acid protein kinase 1) plays important protein kinase activity and ATP binding. A recent study demonstrated that missense *de novo* variants in *TAOK1* cause neurodevelopmental delays in children [53]. The same study showed knockdown of *TAOK1* caused early lethality in the Drosophila.

To confirm our findings, we conducted several validation and confirmatory analyses. First, we compared our data to a population of healthy controls (gnomAD). Rare (gnomAD AF<0.006) compound heterozygous SNVs in *TM2D1*, *MUC16* and *VWA5B2* genes identified in our data were not observed as homozygotes in healthy gnomAD controls or live births. This finding suggested that variants in haplosufficient genes may contribute to fetal demise in offspring of two healthy parent carriers. Given that our filtering approach is cantered on allele frequencies and predicted impact, and is agnostic to the phenotype of interest, the identification of gene candidates associated with congenital and developmental phenotypes is notable. Although we demonstrated some sharing of SNVs across families (e.g., compound heterozygous SNVs in four *TM2D1*, *MUC16*, *VWA5B2* were shared across two families), losses may not have common etiologies [22, 54]. As such, this finding suggests that different genes may play a role at different developmental epochs and across families [16]. Specifically, *TM2D1* (beta-amyloid peptide binding protein) plays a role in G protein-coupled receptor signaling pathway [36]. In mice, regulator of G protein signaling 2 plays critical role in functional remodeling of uterine arteries to impact uterine blood flow during pregnancy [55]. Heterozygous mutations of *TM2D1* and their possible roles in pregnancy loss have not been previously identified. Similarly, *VWA5B2* (von Willebrand factor A domain containing 5B2), with unknown biological function, may play a role in Usher Syndrome Type 1f [36], but its role in pregnancy loss is as yet unknown. However, *MUC16* (Mucin 16) is a glycoprotein involved in cell adhesion. Its expression was found to be reduced in recurrent miscarriage [56]. *MUC16* is considered an inhibitor of implantation [57, 58], underscoring the relevance of compound heterozygous SNVs we identified in fetal death. Furthermore, we explored validation by Sanger sequencing of *VWA5B2*, potentially novel candidate gene little known in the literature. Sanger sequencing confirmed that WGS in our sample confidently called its compound heterozygous SNV (chr3:184236380:T:C). However, further interpretations from our Sanger sequencing results were hindered by the DNA extraction quality and require sequencing of additional samples with higher DNA quality.

Compound heterozygous variants have been previously implicated in pregnancy loss [59] and present a scenario in which each parent is purportedly healthy but carries variants in the same gene(s) that may be incompatible with life. As such, functional validation of inherited compound heterozygous variants may provide a clearer picture of the genetic landscape of recurrent pregnancy loss, especially recurrent cases. *De novo* variants in highly conserved or constrained genes also may lead to pregnancy loss. However, a *de novo* mutation has a much lower recurrence rate than recessive or dominant inherited disorders [60, 61]. Impactful X-linked recessive variants, for example, a missense X-linked SNV (chrX:108591181:C:A) impacting *COL4A5* (Alport syndrome 1 gene) and possibly relevant to fetal death (**S1 File** and **S2 Table**), may also serve as candidates for validation. However, X-linked dominant mutations can also be lethal in male fetuses and need to be further elucidated in larger studies. Importantly, genetic diagnoses based on impactful variants following various modes of inheritance may be used to provide a prognosis based on data from other families with similar mutations [62, 63]. Confirmation of genes relevant to pregnancy loss will also identify critical pathways and novel therapeutic targets for improving pregnancy outcomes.

Our study has several limitations. The higher counts of *de novo* SNVs we observed in pregnancy losses compared with live births could result from sequencing error, reflected from degradation of placenta samples due to FFPE. FFPE samples have small fragment sizes and very uneven coverage, contributing to false positive SNVs/SVs. For example, low quality libraries (high DNA degradation) from two samples may have contributed to the large number of *de novo* SNVs observed in losses in our data. To validate SNVs in our data, we conducted exploratory Sanger sequencing analysis. Results showed poor validation for *de novo* **S1 File** but confirmed several compound heterozygous calls (**Table 3**) that were not confidently called in our samples. Furthermore, we used Slivar, a method that is strictly a filtering strategy, and the utility of the output relies on high-quality input variants. Future studies utilizing freshly obtained placenta samples for WGS may address elevated sequencing error potentially contributed by FFPE.

Strengths of our study include prospective collection of samples from losses and live births, where DNA samples from both parents and liveborn children were available. This may improve strategies for determining the ‘intolerome’, conditions incompatible with life resulting in fetal demise, and potential to improve database of lethal genes and phenotypes, which are poorly represented. Although our study is underpowered to compare rates of SNVs/SVs between losses and live births, our study serves as a requisite feasibility step in exploring genes relevant to pregnancy loss. Thus, the findings from our pilot study will provide justification for conducting WGS using larger parent-offspring families with potential to identify SNVs causing pregnancy loss.

## Conclusion

The findings reported herein provide evidence for genetic variants (including several in previously recognized genes) relevant to unexplained pregnancy loss in families. WGS of DNA from larger numbers of families (including parent-offspring DNA from affected and unaffected pregnancies) may help identify lethal genes contributing to sporadic and recurrent pregnancy loss. Elucidating pregnancy loss causing genes may lead to biomarkers useful for risk stratification, the identification of genes relevant to normal and abnormal pregnancy, and novel therapeutic targets.

## Supporting information

S1 Table Number (percent) of all Slivar prioritized SNVs in products of conception by pathogenicity and mode of inheritance. ^a^P-values are from two-sided 1-degree of freedom Chi-squared test, comparing the proportions of SNVs between losses and live births. **Note**: The denominator is the total Slivar-picked autosomal dominant, *de novo* and compound heterozygous SNVs.
(DOCX)

S2 Table SNVs with high pathogenicity scores (pLI>0.9; LOUEF<0.36) identified in pregnancy losses but not live births. (DOCX)

S3 Table Pathogenicity and loss-of-function *de novo* SNVs in products of conception. ^a^The observed mean *de novo* loss-of-function SNVs in pregnancy losses was higher than that of the expected (2 vs 0.2; p-value = 0.01); SNVs were enriched in >1 protein altering genes (p-value<0.001).
(DOCX)

S1 File (DOCX)
