# Supplementary material for: Whole-genome sequencing analysis in families with recurrent pregnancy loss: A pilot study
Source: PLoS One. 2023 Feb 17;18(2):e0281934. doi: 10.1371/journal.pone.0281934 (PMC9937472; doi:10.1371/journal.pone.0281934)
Supplement: S1 Table — aP-values are from two-sided 1-degree of freedom Chi-squared test, comparing the proportions of SNVs between losses and live births. Note: The denominator is the total Slivar-picked autosomal dominant, de novo and compound heterozygous SNVs. (DOCX) [file pone.0281934.s001.docx]

| **Mode of Inheritance** | **Pregnancy loss** | | | **Live birth** | | | **Total** | ***p-value***^a^ |  |
| --- | --- | --- | --- | --- | --- | --- | --- | --- | --- |
|  | **Total** | **pLI > 0.9; LOEUF < 0.35** | **pLI < 0.9; LOEUF > 0.35** | **Total** | **pLI > 0.9; LOEUF < 0.35** | **pLI < 0.9; LOEUF > 0.35** |  |  |  |
| Autosomal Dominant, n (%) | 296 (47.1) | 56  (8.9) | 240  (38.2) | 332 (52.9) | 68  (10.8) | 264 (42.0) | 628 (100.0) | 0.15 |  |
|  |  |  |  |  |  |  |  |  |  |
| *De novo*, n (%) | 108 (92.3) | 22  (18.8) | 86  (73.5) | 9  (7.7) | 2  (1.7) | 7  (6.0) | 117 (100.0) | <0.001 |  |
|  |  |  |  |  |  |  |  |  |  |
| Compound Heterozygous, n (%) | 27 (42.2) | 5  (7.8) | 22  (34.4) | 37 (57.8) | 5  (7.8) | 32  (50.0) | 64 (100.0) | 0.21 |  |
|  |  |  |  |  |  |  |  |  |  |
| **Total, n (%)** | **432 (53.3)** | **84**  **(10.4)** | **348**  **(43.0)** | **378 (46.7)** | **75**  **(9.3)** | **303 (37.4)** | **810 (100.0)** | **0.06** |  |
|  |  |  |  |  |  |  |  |  |  |
