# Supplementary material for: Whole-genome sequencing analysis in families with recurrent pregnancy loss: A pilot study
Source: PLoS One. 2023 Feb 17;18(2):e0281934. doi: 10.1371/journal.pone.0281934 (PMC9937472; doi:10.1371/journal.pone.0281934)
Supplement: S2 Table — (DOCX) [file pone.0281934.s002.docx]

| **Family** | **Outcome** | **SNV** | **cDNA position** | **Mode of Inheritance** | **Highest Impact** | **Gene** | **Nomenclature** | **Biological Function ^a^** | **Associated Diseases** |
| --- | --- | --- | --- | --- | --- | --- | --- | --- | --- |
| Family 1 | fetal death | chr3:63912887:C:A | 289 | Autosomal Dominant | Missense | *ATXN7* | Ataxin 7 | Protein binding | Spinocerebellar ataxia 7 |
|  | fetal death | chr3:63912887:C:A |  |  |  |  |  |  |  |
|  | fetal death | chr19:17314331:C:A | - | Autosomal Dominant | Splice region | *DDA1* | DET1- and DDB1-associated protein 1; placenta cross-immune reaction antigen 1 | Protein binding | - |
|  | stillbirth | chr14:95129520:ATTTTCTCTAGTTTCTGAATC:A | 686 | *de novo* | Frameshift | *DICER1* | dicer 1, ribonuclease III | Nucleotide, DNA and RNA binding | Embryonal; Global developmental delay |
|  | stillbirth | chr10:419184:G:A | 620 | Autosomal Dominant | Missense | *DIP2C* | Disco Interacting Protein 2 Homolog C | Molecular function | - |
|  | fetal death |  |  |  |  |  |  |  |  |
|  | fetal death |  |  |  |  |  |  |  |  |
|  | fetal death | chr9:83970202:G:A | 1321 | *de novo* | Stop gained | *HNRNPK* | Heterogeneous Nuclear Ribonucleoprotein K | Nucleic acid, RNA and protein binding | Au-Kline Syndrome |
|  | fetal death | chr10:17840773:C:T | 883 | Autosomal Dominant | Missense | *MRC1* | Mannose Receptor C-Type 1 | Mannose, protein binding | Gaucher's Disease |
|  | stillbirth | chr3:88139246:C:T | 755 | *de novo* | Missense | *ZNF654* | Zinc Finger Protein 654 | DNA-binding transcription factor activity, RNA polymerase II-specific | - |
| Family 4 | fetal death | chrX:108591181:C:A | 1289 | X-linked recessive | Missense | *COL4A5* | Alport syndrome 1 | Extracellular matrix structural constituent | Alport syndrome 1 |
|  | fetal death | chr11:117458790:G:A | 3532 | Autosomal Dominant | Missense | *DSCAML1* | DS Cell Adhesion Molecule Like 1 | Protein homodimerization activity and cell-cell adhesion mediator | Down Syndrome |
|  | fetal death | chr5:128335211:TAGCAGAGGCAGCGATACTCTCCAGGAATGTTGGTACACTGGCCGCCATCAC:T | 3932 | *de novo* | Inframe Deletion | *FBN2* | Fibrillin-2 | Extracellular matrix structural constituent | Congenital contractural arachnodactyly |
|  | fetal death | chr8:41977214:C:T | 1157 | *de novo* | Missense | *KAT6A* | Lysine Acetyltransferase 6A | Regulation of DNA-templated transcription | Mental retardation, autosomal dominant 32 |
|  | fetal death | chr9:33062105:CTGCCTTGCCTCGAAACAAATCTATGGTCATACCAGGAGGAAGCAATCCCTGATGCTGCTGCCACTTCAG:C | 574 | *de novo* | Inframe Deletion | *SMU1* | SMU1 DNA Replication Regulator And Spliceosomal Factor | Protein binding | - |
| Family 3 | embryonic-loss | chr7:140739949:GGA:G | - | *de novo* | Splice region | *BRAF* | B-Raf Proto-Oncogene, Serine/Threonine Kinase | Protein kinase activity | Noonan syndrome 7 |
|  | stillbirth | chr6:36200959:G:A | 637 | *de novo* | Missense | *BRPF3* | Bromodomain and PHD finger-containing protein 3 | Protein binding | - |
|  | embryonic-loss | chr17:47171999:C:T | 169 | *de novo* | Missense | *CDC27* | Cell Division Cycle 27 | Protein phosphatase binding | - |
|  | fetal death | chr5:180603262:G:A | 4022 | Autosomal Dominant | Missense | *FLT4* | Fms Related Receptor Tyrosine Kinase 4 | Transmembrane receptor protein tyrosine kinase activity; vascular endothelial growth factor receptor activity | Congenital heart defects |
|  | embryonic-loss | chr15:63774800:G:T | 824 | *de novo* | Stop gained | *HERC1* | HECT And RLD Domain Containing E3 Ubiquitin Protein Ligase Family Member 1 | Ubiquitin-protein transferase activity | Macrocephaly |
|  | embryonic-loss | chr15:63774801:A:C | 823 | *de novo* | Missense |  |  |  |  |
|  | embryonic-loss | chr15:63774805:C:CT | 819 | *de novo* | Frameshift |  |  |  |  |
|  | embryonic-loss | chr5:150134013:TAG:T | - | *de novo* | Splice region | *PDGFRB* | Kosaki overgrowth syndrome | Protein tyrosine kinase activity; platelet activating factor receptor activity | Kosaki overgrowth syndrome |
|  | embryonic-loss | chr15:43766828:TTGGAGAGCACTGC:T | 947 | *de novo* | Frameshift | *PDIA3* | Protein Disulfide Isomerase Family A Member 3 | Protein and RNA binding | Prion Disease |
|  | embryonic-loss | chr8:140668348:G:A | 2786 | *de novo* | Missense | *PTK2* | Protein Tyrosine Kinase 2 | Protein tyrosine kinase activity | Malignant Astrocytoma; Ovarian Cancer |
|  | embryonic-loss | chr9:127069303:G:A | 557 | *de novo* | Missense | *RALGPS1* | Ral GEF With PH Domain And SH3 Binding Motif 1 | Guanyl-nucleotide exchange factor activity | Developmental And Epileptic Encephalopathy 4; Uterine Carcinosarcoma |
|  | stillbirth | chr2:11249767:G:A | 356 | *de novo* | Missense | *ROCK2* | Rho Associated Coiled-Coil Containing Protein Kinase 2 | Protein serine/threonine kinase activity | Ureteral Obstruction |
|  | embryonic-loss | chr12:27309142:G:A | 338 | *de novo* | Missense | *STK38L* | Serine/Threonine Kinase 38 Like | Protein serine/threonine kinase activity; magnesium ion binding | Macular Degeneration |
|  | stillbirth | chr6:147316370:TC:T | 1765 | *de novo* | Frameshift | *STXBP5* | Syntaxin Binding Protein 5 | GTPase activator activity; syntaxin and myosin II binding | Von Willebrand Disease |
|  | stillbirth | chr17:29507963:G:A | 1406 | *de novo* | Missense | *TAOK1* | Thousand And One Amino Acid Protein Kinase 1 | Protein kinase activity and ATP binding | Developmental Delay With Or Without Intellectual Impairment |
|  | fetal death | chr2:169994355:A:AG | 3817 | *de novo* | Frameshift | *UBR3* | Ubiquitin Protein Ligase E3 Component N-Recognin 3 | Ubiquitin protein ligase activity | - |
|  | stillbirth | chr2:61227175:TCTTCTTCTTCCC:T | 7487 | *de novo* | Inframe Deletion | *USP34* | Ubiquitin Specific Peptidase 34 | Cysteine-type endopeptidase activity; protein binding | - |
