# Supplementary material for: Whole-genome sequencing analysis in families with recurrent pregnancy loss: A pilot study
Source: PLoS One. 2023 Feb 17;18(2):e0281934. doi: 10.1371/journal.pone.0281934 (PMC9937472; doi:10.1371/journal.pone.0281934)
Supplement: S3 Table — aThe observed mean de novo loss-of-function SNVs in pregnancy losses was higher than that of the expected (2 vs 0.2; p-value = 0.01); SNVs were enriched in >1 protein altering genes (p-value<0.001). (DOCX) [file pone.0281934.s003.docx]

| **Variant Classification** | **Products of Conception** | | | | | |
| --- | --- | --- | --- | --- | --- | --- |
|  | **Pregnancy loss** ^a^ | | | **Live birth** | | |
|  | pLI > 0.9; LOEUF < 0.35 | pLI < 0.9; LOEUF > 0.35 | **Total** | pLI > 0.9; LOEUF < 0.35 | pLI < 0.9; LOEUF > 0.35 | **Total** |
| In-frame Deletion/Insertion | 3 | 7 | **10** | 0 | 0 | **0** |
| Missense | 10 | 36 | **46** | 2 | 4 | **6** |
| Frameshift | 5 | 18 | **23** | 0 | 0 | **0** |
| Stop gained | 2 | 6 | **8** | 0 | 0 | **0** |
| Splice region | 2 | 19 | **21** | 0 | 3 | **3** |
| **Total** | **22** | **86** | **108** | **2** | **7** | **9** |
