# Supplementary material for: Whole-genome sequencing analysis in families with recurrent pregnancy loss: A pilot study
Source: PLoS One. 2023 Feb 17;18(2):e0281934. doi: 10.1371/journal.pone.0281934 (PMC9937472; doi:10.1371/journal.pone.0281934)
Supplement: S1 File — (DOCX) [file pone.0281934.s004.docx]

**Whole-genome sequencing analysis in families with recurrent pregnancy loss: A pilot study**

Tsegaselassie Workalemahu^1^, Cecile Avery^2^, Sarah Lopez^1^, Nathan R. Blue^1,3^, Amelia Wallace^2^, Aaron R. Quinlan^2,5^, Hilary Coon^4^, Derek Warner^7^, Michael W. Varner^1,3^, D. Ware Branch^1,3^, Lynn B. Jorde^2^, Robert M. Silver^1,3^

^1^University of Utah Health, Department of Obstetrics and Gynecology, Salt Lake City, Utah, United States

^2^University of Utah, Department of Human Genetics, Salt Lake City, Utah, United States

^3^Intermountain Healthcare, Maternal-Fetal Medicine, Salt Lake City, Utah, United States

^4^University of Utah, Department of Psychiatry, Salt Lake City, Utah, United States

^5^Department of Biomedical Informatics, University of Utah, Salt Lake City, United States

^7^DNA Sequencing Core, University of Utah, Salt Lake City, UT, USA

**Corresponding author**: Tsegaselassie Workalemahu, PhD MS; University of Utah, 30 North 1900 East, Suite 2B293, Salt Lake City, UT, 84132. Phone: 801-587-1607. Email: tsegaselassie.workalemahu@hsc.utah.edu

**Online supplement methods**

**Variant detection and quality control of WGS**

Whole-genome analysis was performed by the Utah Center for Genomic Discovery (UCGD) at the University of Utah [1]. Germline SNVs and SVs for each sample (22 samples total) were detected following a Genome Analysis Tool Kit (GATK) best practices equivalent workflow for variant detection. First, raw data output short reads were aligned using the GRCh38 human reference genome, using alt-aware alignment and variant calling against the GRCh38 build with alt and decoy contigs. The pipeline included FastQForward, an in-house multiprocess-parallelization manager that wraps Burrows-Wheeler Alignment (BWA-MEM) for sequence alignment [2], SAMBLASTER for duplicate read marking [3], Sambamba for data manipulation [4], and Sentieon Haplotyper and GVCFtyper for variant identification and joint genotyping. Next, the variant effect predictor was used to annotate called variants [5]. Finally, data quality control (QC) reports were generated from fastp [6], indexcov [7], Alignstats (https://github.com/jfarek/alignstats), and BCFtools stats [8], and were aggregated with MultiQC [9]. Peddy was used to check ancestry, sex, and family relationships [10]. Phenotype-specific candidate genes were checked for sufficient sequence depth using seqcover (https://github.com/mikecormier/neoseq-seqcover-nf). We increased the BWA align seed from its default 19 to 24 to effectively prevent bacterial reads in saliva samples from mismapping to the human reference.

To filter potentially false-positive genotype calls, we used genotype quality (GQ), a Phred-scaled value representing the confidence that the called genotype is the true genotype. Values representing the number of reads passing QC were used to calculate the genotype at a specific site in a specific sample. Allele balance (AB), the ratio of reads aligned at a variant locus that support the alternate allele filtering, metrics were generated by the Sentieon tools (a commercial pipeline that is equivalent to GATK) [11]. We selected variants with GQ≥20 and AB between 0.2 and 0.8, the recommended cut-offs for data with low sequencing coverage [12]. Additional filtering was performed based on a Genome Aggregation Database (gnomAD [13]) allele frequency (AF)<0.001 and whether a variant was predicted to have a higher predicted impact on gene function (e.g., stop gained, frameshift, missense). Variant detection methods were tuned to detect low-frequency mutations (gnomAD allele frequency AF<0.001) to explore and compare germline variants in protein coding regions (potentially impactful variants) across samples.

**Prioritization of SNVs by pathogenicity and functional impact in genes**

We used Slivar [12], variant prioritization tool developed by our team. Slivar utilizes phasing-by-inheritance, in which each non-reference variant has been identified as maternal or paternal, given the parental genotype to identify pathogenic candidate variants and allow filtering by inheritance pattern. In addition, Slivar utilizes a pedigree (.ped) file representing all individuals in the variant call file (.vcf), and a set of custom filtering expressions as input. This includes setting the maximum population AF> 0.001, retaining variants annotated for functional regions of the genome including exons, splice sites, regulatory regions, and variant quality metrics.

For each SNV, Slivar assigned pathogenicity scores by using the probability of being loss-of-function intolerant (pLI) and the loss-of-function observed/expected upper bound fraction (LOEUF) [14, 15]. We used pLI>0.90 and LOEUF<0.36 for selection of SNVs with high pathogenicity scores [16]. In addition, we utilized Slivar to obtain data from ClinVar [17], which provided a collection of published reports of associations between genes and clinically relevant phenotypes.

**Exploratory statistical analyses, SV prioritization and Sanger sequencing**

**Statistical analysis of de novo SNVs**

Given the limited sample size, we explored enrichment of *de novo* SNVs in pregnancy losses combining embryonic loss, fetal death and stillbirth. We used Denovolyze [18], an R function that calculates *de novo* SNV enrichment by dividing the observed number of *de novo* SNVs over the expected number, to determine whether there were more *de novo* SNVs than expected in pregnancy losses (n=6). However, due to the limited sample size in this pilot study, we would be underpowered to detect an excess of coding DNMs by variant class throughout the entire genome and by variant class in individual genes. Therefore, using the R function, we explored whether there may be excess of genes with >1 loss of function SNVs and >1 protein altering SNVs. Analyses were performed using Slivar and R utilizing resources and support from the Center for High Performance Computing at the University of Utah.

**Comparison of SNV rates**

To compare SNV rates by modes of inheritance and SNV impact between losses and live births for each family, we used the two-sided Poisson exact rate ratio test. We determined p-values from the test following a binomial distribution. Furthermore, we compared embryonic loss, fetal death and stillbirth with live births, individually, to compare losses that may have distinct etiologies [19, 20]. In addition, assuming that rates of SNVs will be different across families [21], we compared the SNV rates for each family.

**Rare-variant association testing**

We used the Pedigree Variant Annotation, Analysis, and Search Tool (pVAAST) that applies a composite likelihood ratio test (CLRT) [22] to conduct rare-variant association testing. pVAAST provides a linkage-association score and a p-value for each evaluated gene and, a score for each variant carried by the gene to help further prioritize rare variants. The scores generated by pVASST provide aggregated variant level evidence to produce a rank order list of genes with higher genetic burden in cases than controls. Three pedigrees were analysed with an ancestry matched controls consisting of 1000 Genomes European-ancestry (EUR) and the Utah Centre d′Etudes du Polymorphisme Humain (CEPH; n=287) data. Four WGS losses representing three families were utilized for this method to minimize type 1 error.

**SV prioritization**

We used LUMPY-smoove [23], a probabilistic framework for SV detection and filtering SVs based on spurious alignment signals. For each SV, scores representing depth changes within events and at the break-points (Duphold) [24] were added to SV calls to the estimate the detection accuracy. SV allele frequencies were annotated using SVAFotate, which was used to add SV allele frequencies from gnomAD and 1000 Genome to enable filtering for rare and novel SVs, and SVs that intersect with previously implicated pregnancy loss genes. Furthermore, SVs were filtered on inheritance type, SV type, impact, and duphold flank fold-change (DHFFC) scores (deletions: DHFFC < 0.7, duplications: DHFBC > 1.25) using SVAFotate. Slivar was used to filter SVs on inheritance patterns. And were additionally filtered on a size no larger than 1 Mb. This analysis is based on a single SV calling tool, LUMPY-smoove. A more thorough investigation would include RUFUS and Manta calls to produce a set of consensus SV calls.

**Sanger sequencing**

Confirmatory sequencing of candidate variants was performed via Sanger sequencing on each sample in the pedigree to establish presence or absence of the variant and establish mode of transmission. Primers were designed to target a region of 200-800 base pairs encompassing the variant of interest. Primers were unique to a single location in the genome, have a GC content 40-60%, and contain a GC clamp. High-quality cell line DNA was used as a positive control to determine the efficiency of the primers. Double coverage was achieved by sequencing the forward and reverse strand. Sequencing traces were aligned, trimmed, and visualized with Sequencher [25].

**Online supplement results**

**WGS quality control and variant detection**

Target coverage for the samples varied (sequence coverage fold-mean range: 23-68X, average: 47X; sequence coverage fold-median range: 10-58X, average 36X) after removing low-quality sequences and PCR duplications. MultiQC reports showed variable but good overall base quality across samples for eight parental, ten placental and ten live-birth DNA samples, for twenty-two samples in total ( **Table 1**). The overall QC suggested DNA isolation, sequencing, and analytic pipeline performed as expected for most samples. However, mean WGS coverage of two FFPE samples in Family 1 was higher (>30X), suggesting non-uniform coverage (e.g., short fragments and poor DNA/library). WGS coverage ranged from poor to high coverage (i.e., 31% to 91%). Two FFPE samples from Trio 1 and Trio 3 were outliers showing low coverage. Duplicate reads ranged from 16% to-25%, values considered higher for PCR-free libraries. Unmapped reads ranged from 0.17% to-44%, which suggested high variability, possibly due to bacterial content of saliva samples. High values in soft clipped bases (range: 0.9-13.1%) suggested FFPE degradation and read-through adapter content fragments <150 bp. Low mean insert sizes (range: 150-485) and variability also reflected FFPE degradation. The heterozygosity rates for two FFPE samples in Family 1 and two FFPE samples from Family 3 were very low, and the heterozygosity rate for the frozen placenta in Family 1 was very high, suggesting genome-wide DNA contamination. Sex-check revealed that Family 2 FFPE sample was identical to the mother’s DNA, indicating severe maternal cell contamination. Finally, relationship-check showed atypical relatedness in Family 2, possibly due to artifacts in FFPE degradation.

The quality of FFPE-based WGS data was noticeably different from those of saliva-based WGS. For example, FFPE-based WGS data had very low fragment sizes with smaller insert sizes (200bp) after library prep for Family 3, which suggested degradation of input DNA. However, this was not uniformly observed across all FFPE-based WGS data. After applying quality control, we included eight samples from losses and families (Family 1, Family 3 and Family 4) that had at least one representative FFPE sample.

FFPE-derived WGS also tended to have a higher proportion of missing genotypes. Excess heterozygosity indicating potential maternal cell contamination has been observed in pregnancy loss sample-based WGS[26] and is likely due to the nature of sampling the placenta. Samples that fell 3 ± standard deviations from the mean heterozygosity were excluded from Slivar and pVAAST analyses but retained for follow-up of candidate variants. The transition/transversion ratio (ts/tv) across samples was approximately 2.0, which is typical for WGS data.[27] Noticeably, the FFPE samples with lower quality libraries had ts/tv ratios around 1.8, indicating a higher false positive rate. To address this, candidate variants were reviewed manually via Integrated Genome Viewer (IGV) to investigate allele balance and variant quality. Sanger sequencing was also performed except when DNA quality was prohibitive. We excluded Family 2 from our interpretations of the WGS data because the stillbirth FFPE placenta, the only product of conception from losses available in Family 2, had >15% genotype call error rate and excess mean heterozygosity (**Table 1**). Out of 19,498,680 SNVs, 11,296,578 SNVs remained after variant filtering. After removing poor DNA quality samples and samples failing sex-check (five pregnancy losses samples and one family), 3,211,893 SNVs remained for further analysis. Finally, 28,485 impactful SNVs (i.e., missense, frameshift, insertion/deletion, stop gained/retained, and splice region) in all samples from the products of conception (n=16 losses and live births in three families) were prioritized by Slivar.

**Exploratory SNV rates comparison analysis**

The total count of SNVs among eight losses across the families was higher than that of seven live-births (53.3% vs. 46.7%, respectively; p-value=0.06; **S1 Table**). Specifically, the proportion of *de novo* SNVs was higher in losses than live births (92.3% vs. 7.7%, respectively; p-value<0.001). However, the proportions of SNVs following autosomal dominant and compound heterozygous modes of inheritance, were higher in live births than losses (52.9% vs. 47.1%; p-value=0.15 and 57.8% vs. 42.2%; p-value=0.21, respectively).

**Exploratory rare-variant association testing**

We performed exploratory pVAAST analysis of cases (n=4 pregnancy losses) and ancestry-matched controls (n=287 1000G EUR and CEPH population) and interrogated variants that fit an autosomal recessive mode of inheritance in genes (n=20,004) in our samples. LOD scores ranged between 0 and 1.83, and the gene burden scores ranged between 0 and 39.4. Given the presumed genetic heterogeneity of pregnancy loss and number of samples excluded from the analysis due to quality control, the rare-variant association testing was statistically underpowered. Although we observed several variants that were ranked from highest to lowest gene burden with p-values<0.05, the top candidates gene included variants that were flagged as potential false positives. This was confirmed in gnomAD when the variants failed quality score recalibration filter. Manual investigation of these variants in all sampled family members via IGV revealed that these variants were often Mendelian violations, showing skewed allele balance, likely due to a small fraction of false positive variant calls in sequenced reads (data not shown). Thus, this exploratory analysis was excluded from our interpretations but confirmed several false positive autosomal recessive variants prioritized in our main analysis findings.

**Exploratory SV prioritization**

SV calls were intersected against Slivar SNVs to either 1) generate SV candidates that might produce a more compelling compound heterozygous pathogenic variant than identified by looking at SNVs alone, or 2) produce a new compound heterozygous hits from an ‘autosomal dominant’ candidate SNV, in which, one side of the compound heterozygous hit is a SNV and the other an SV. The resulting SV list varied per family and ranged from three candidates to nearly twenty.

In addition, *de novo* SVs were intersected with a list of curated RPL genes. Several SVs annotated to impact recurrent pregnancy loss genes were shared between no more than two families. However, many of these events are likely false positives. A subset of distinct SVs that fell within the same annotated gene between families were investigated manually on IGV. Samples representing the SV as well as parental samples were used to visually confirm the presence and type of SV. While reads supporting the event were found in samples, those reads are not abundant and often neighbour other split read pairs. These regions are not problematic in parental samples, and the patterns exhibited in losses are likely due to DNA degradation. Degradation of samples provides a challenge for SV calling and interpretation, and these results are largely inconclusive. It possible that <1 Mb SVs contribute to recurrent pregnancy loss, but sequencing strategies or highly stringent filtering criteria designed to limit excess false positives are necessary.

**Exploratory Sanger sequencing analysis**

Sanger sequencing effectively eliminated several false positive *de novo* variants observed in losses. However, some loci were less tractable to confirmatory sequencing, even in positive control samples. Despite passing all design metrics, several candidate *de novo* loci including those targeting *KAT6A* and *SMU1* genes showed non-specific PCR amplification not conducive to Sanger sequencing. These genomic locations do not appear to be problematic in larger control datasets, but these variants remain speculative. Sanger sequencing was able to confirm several compound heterozygous calls, e.g., those targeting *VWA5B2* gene, and was informative to the genotype of losses that were not confidently called in WGS. In some cases, DNA quality was not amenable to Sanger sequencing. The effect of maternal cell contamination could be observed by the difference in peak intensity of heterozygous positions in which the maternal allele was the dominant signal. The results from Sanger sequencing were limited by the quality of the initial DNA extraction.

**References**

1. Franke KR, Crowgey EL. Accelerating next generation sequencing data analysis: an evaluation of optimized best practices for Genome Analysis Toolkit algorithms. Genomics & informatics. 2020;18(1).

2. Li H. Aligning sequence reads, clone sequences and assembly contigs with BWA-MEM. arXiv preprint arXiv:13033997. 2013.

3. Faust GG, Hall IM. SAMBLASTER: fast duplicate marking and structural variant read extraction. Bioinformatics. 2014;30(17):2503-5.

4. Tarasov A, Vilella AJ, Cuppen E, Nijman IJ, Prins P. Sambamba: fast processing of NGS alignment formats. Bioinformatics. 2015;31(12):2032-4.

5. McLaren W, Gil L, Hunt SE, Riat HS, Ritchie GR, Thormann A, et al. The ensembl variant effect predictor. Genome biology. 2016;17(1):1-14.

6. Chen S, Zhou Y, Chen Y, Gu J. fastp: an ultra-fast all-in-one FASTQ preprocessor. Bioinformatics. 2018;34(17):i884-i90.

7. Pedersen BS, Collins RL, Talkowski ME, Quinlan AR. Indexcov: fast coverage quality control for whole-genome sequencing. Gigascience. 2017;6(11):gix090.

8. Li H. A statistical framework for SNP calling, mutation discovery, association mapping and population genetical parameter estimation from sequencing data. Bioinformatics. 2011;27(21):2987-93.

9. Ewels P, Magnusson M, Lundin S, Käller M. MultiQC: summarize analysis results for multiple tools and samples in a single report. Bioinformatics. 2016;32(19):3047-8.

10. Pedersen BS, Quinlan AR. Who’s who? Detecting and resolving sample anomalies in human DNA sequencing studies with peddy. The American Journal of Human Genetics. 2017;100(3):406-13.

11. Carson AR, Smith EN, Matsui H, Brækkan SK, Jepsen K, Hansen J-B, et al. Effective filtering strategies to improve data quality from population-based whole exome sequencing studies. BMC bioinformatics. 2014;15(1):1-15.

12. Pedersen BS, Brown JM, Dashnow H, Wallace AD, Velinder M, Tristani-Firouzi M, et al. Effective variant filtering and expected candidate variant yield in studies of rare human disease. NPJ Genomic Medicine. 2021;6(1):1-8.

13. Karczewski K, Francioli L, Tiao G, Cummings B, Alföldi J, Wang Q. Genome Aggregation Database, C.(2020). The mutational constraint spectrum quantified from variation in. 141:434-43.

14. Samocha KE, Robinson EB, Sanders SJ, Stevens C, Sabo A, McGrath LM, et al. A framework for the interpretation of de novo mutation in human disease. Nature genetics. 2014;46(9):944-50.

15. Wang W, Corominas R, Lin GN. De novo mutations from whole exome sequencing in neurodevelopmental and psychiatric disorders: from discovery to application. Frontiers in genetics. 2019;10:258.

16. Karczewski KJ, Francioli LC, Tiao G, Cummings BB, Alföldi J, Wang Q, et al. The mutational constraint spectrum quantified from variation in 141,456 humans. Nature. 2020;581(7809):434-43.

17. Landrum MJ, Lee JM, Riley GR, Jang W, Rubinstein WS, Church DM, et al. ClinVar: public archive of relationships among sequence variation and human phenotype. Nucleic acids research. 2014;42(D1):D980-D5.

18. Ware JS, Samocha KE, Homsy J, Daly MJ. Interpreting de novo variation in human disease using denovolyzeR. Current protocols in human genetics. 2015;87(1):7.25. 1-7.. 15.

19. Blue NR, Page JM, Silver RM, editors. Genetic abnormalities and pregnancy loss. Seminars in Perinatology; 2019: Elsevier.

20. Silver RM, Branch DW, Goldenberg R, Iams JD, Klebanoff MA. Nomenclature for pregnancy outcomes: time for a change. Obstetrics & Gynecology. 2011;118(6):1402-8.

21. Kline J, Vardarajan B, Abhyankar A, Kytömaa S, Levin B, Sobreira N, et al. Embryonic lethal genetic variants and chromosomally normal pregnancy loss. Fertility and sterility. 2021;116(5):1351-8.

22. Yandell M, Huff C, Hu H, Singleton M, Moore B, Xing J, et al. A probabilistic disease-gene finder for personal genomes. Genome research. 2011;21(9):1529-42.

23. Layer RM, Chiang C, Quinlan AR, Hall IM. LUMPY: a probabilistic framework for structural variant discovery. Genome biology. 2014;15(6):R84.

24. Pedersen BS, Quinlan AR. Duphold: scalable, depth-based annotation and curation of high-confidence structural variant calls. Gigascience. 2019;8(4):giz040.

25. Nishimura D. Sequencher 3.1. 1. Biotech Software & Internet Report. 2000;1(1-2):24-30.

26. Rajcan-Separovic E. Next generation sequencing in recurrent pregnancy loss-approaches and outcomes. European Journal of Medical Genetics. 2020;63(2):103644.

27. DePristo MA, Banks E, Poplin R, Garimella KV, Maguire JR, Hartl C, et al. A framework for variation discovery and genotyping using next-generation DNA sequencing data. Nature genetics. 2011;43(5):491-8.
